# Editor’s Corner: Negative Trials and Tribulations

**DOI:** 10.1080/24740527.2019.1670494

**Published:** 2019-10-18

**Authors:** Joel Katz

**Affiliations:** Editor-in-Chief, *Canadian Journal of Pain*, Department of Psychology, York University, Toronto, Canada; Department of Anesthesia and Pain Management, Toronto General Hospital, Toronto, Canada

From time to time the *Canadian Journal of Pain* will consider publishing reports of clinical trials that were discontinued at some point during the course of the trial. Readers may be surprised to learn that between 30% and 50% of clinical trials never get published.^1,2^ This applies to the adult^2^ and pediatric^1^ literatures. Reasons for trial discontinuation and non-publication are numerous but the most frequently cited is difficulty recruiting the required sample size.^1,3^ Non-publication results in a dissemination or publication bias^2^ characterized by the presence of nonrandom differences between the published and non-published (gray) literatures.^4^ For example, studies reporting statistically significant/positive trials are twice as likely to get published as those with non-significant/negative findings and effect sizes of published studies are greater than non-published studies or those in the gray literature.^2,4^ The consequences of selective publication include inaccurate results of meta-analyses,^5^ wasted resources,^2^ threat to the integrity of the scientific/medical literature,^6^ and potential harm to patients.^7^ Some authors have even argued that failure to report the results of clinical trials amounts to academic misconduct.^6^

In this issue, Lynch et al.^8^ report the results of a three-site, randomized, double-blind, controlled trial comparing the efficacy of 11 weeks of twice daily oral methadone (5–60 mg per 24 hours) versus controlled release morphine (20–240 mg per 24 hours) capsules for moderate-to-severe chronic neuropathic pain of central or peripheral origin present for 3 months or longer. Other inclusion criteria were a score of 4/10 or more on the Douleur Neuropathique 4 (DN4) pain questionnaire, average 7-day pain intensity score >4/10 on a 0–10 numeric rating scale (NRS), stable levels of concomitant non-opioid analgesics, stable levels of other non-pharmacologic interventions, and, initially, no more than 90 mg in morphine equivalents/day (MME/day). Sample size estimation showed that 67 participants per group would provide enough power (0.96) to detect a 2-point difference in pain intensity on a 0–10 numeric rating scale in favor of methadone. Secondary outcomes included pain interference, quality of life, mood, global impression of change, and adverse effects. Participants were required to attend the clinic on 7 occasions over the course of the 11-week trial. Mid-trial, challenges with recruitment necessitated a research ethics board amendment to increase the MME/day from 90 mg to a maximum of 160 mg. Recruitment spanned a 36-month period at the three sites during which >700 participants were screened for eligibility. Of the 83 eligible participants, only 14 participants were randomized to receive methadone (n = 6) or morphine (n = 8) and all completed the trial. After three years, the trial was discontinued due the challenges recruiting participants within the funding period. The authors refrained from conducting parametric analyses given that they only managed to recruit ~21% of the required sample size and instead report descriptive statistics for the two groups showing the outcomes on the primary and secondary measures. Interested readers can see for themselves the magnitude of changes over time for the participants who received methadone or morphine.

Consistent with the literature,^1,3^ Lynch et al.^8^ report that their inability to recruit the required sample size within the specified time period was the primary reason for discontinuation of the trial. Restrictive inclusion and exclusion criteria as well as the requirement that participants attend 7 clinic visits throughout the study period contributed to the failure to reach the target number of participants. Increasing the MME/day from a maximum of 90 mg to 160 mg did not appreciably improve the accrual rate. While it could be argued that an initial feasibility^9^ or proof of concept^10^ study might have alerted the authors to the participant accrual problem before embarking on an RCT, it is also possible, as the authors argue, that unanticipated changes in the climate of opioid prescribing during the conduct of the trial appear to have contributed to the difficulty recruiting participants.

The study by Lynch et al.^8^ serves to alert future researchers to the challenges in recruiting participants with chronic pain for clinical trials evaluating opioids and at the same time may provide effects sizes for oral methadone and controlled release morphine for those brave enough to embark on a similar study.

## References

[1] Pica N, Bourgeois F. Discontinuation and nonpublication of randomized clinical trials conducted in children. Pediatrics. 2016;138(3):e20160223–e20160223. doi:10.1542/peds.2016-0223. 27492817 PMC5005019

[2] Song F, Parekh S, Hooper L, Loke Y, Ryder J, Sutton A, Hing C, Kwok C, Pang C, Harvey I. Dissemination and publication of research findings: an updated review of related biases. Health Technol Assess. 2010;14(8):Iii,Ix-Xi, 1–193. doi:10.3310/hta14080. 20181324

[3] Huang G, Bull J, Johnston Mckee K, Mahon E, Harper B, Roberts J, Team Crp. Clinical trials recruitment planning: a proposed framework from the clinical trials transformation initiative. Contemp Clin Trials. 2018;66:74–79. doi:10.1016/j.cct.2018.01.003. 29330082

[4] Song F, Hooper L, Loke Y. Publication bias: what is it? how do we measure it? how do we avoid it? Open Access J Clin Trials. 2013;5:71–81. doi:10.2147/OAJCT.S34419.

[5] Murad M, Chu H, Lin L, Wang Z. The effect of publication bias magnitude and direction on the certainty in evidence. BMJ Evid Based Med. 2018;23(3):84–86. doi:10.1136/bmjebm-2018-110891. PMC5969367 29650725

[6] Wallach J, Krumholz H. Not reporting results of a clinical trial is academic misconduct. Ann Intern Med. 2019;171:293. doi:10.7326/M19-1273. 31060050

[7] Every-Palmer S, Howick J. How evidence-based medicine is failing due to biased trials and selective publication. J Eval Clin Pract. 2014;20(6):908–14. doi:10.1111/jep.12147. 24819404

[8] Lynch M, Moulin D, Perez J. Methadone vs. morphine SR for treatment of neuropathic pain: A randomized controlled trial and the challenges in recruitment. Can J Pain. 2019;3(1). doi:10.1080/24740527.2019.1660575 PMC8730636 35005408

[9] Blatch-Jones A, Pek W, Kirkpatrick E, Ashton-Key M. Role of feasibility and pilot studies in randomised controlled trials: a cross-sectional study. BMJ Open. 2018;8(9):E022233. doi:10.1136/bmjopen-2018-022233. PMC6169762 30257847

[10] Gewandter J, Dworkin R, Turk D, Mcdermott M, Baron R, Gastonguay M, Gilron I, Katz N, Mehta C, Raja S, et al. Research designs for proof-of-concept chronic pain clinical trials: immpact recommendations. Pain. 2014;155(9):1683–95. doi:10.1016/j.pain.2014.05.025. 24865794 PMC4500524

